# Influence of filling technique on fracture resistance of giomer-restored MOD-cavities

**DOI:** 10.1186/s12903-025-06790-w

**Published:** 2025-09-19

**Authors:** Ahmed Saad Naser, Dena Safwat Mustafa, Dina Ezzeldin Mohamed

**Affiliations:** 1 Masters Clinical Esthetic Dentistry, Ain Shams University, Cairo, Egypt; 2 Operative Dentistry Department, Faculty of Dentistry, Ain Shams University, Cairo, Egypt; 3 Conservative Dentistry Department, Faculty of Dentistry, Cairo University, Cairo, Egypt

**Keywords:** Layering, Incrementation, Bulk-fill, Clinical performance, Placement technique, Giomer, Packable, Injectable

## Abstract

**Aim:**

This study assessed the fracture resistance of upper premolars based on the placement technique of giomer restoration in MOD cavities.

**Materials and methods:**

Seventy sound maxillary premolars were divided into five groups (n=14). A standardized MOD cavity was prepared in each tooth (2 mm buccolingual width, 2±0.2 mm central floor depth, and 4±0.2 mm proximal depth).

Teeth were assigned to four experimental groups: Group I received Packable material (Beautifil II LS, Shofu Inc, Japan) applied in 2mm increments; Group II received Injectable material (Beautifil Flow Plus X, Shofu); Group III received a packable bulk-fill variant (Beautifil-Bulk Restorative Packable); and Group IV received flowable bulk-fill material (Beautifil-Bulk Flowable, Shofu), each followed by a 2mm occlusal capping layer. A fifth group of intact premolars served as the control.

All specimens were embedded in self-cure acrylic resin blocks with simulated periodontal ligaments at temperatures ranging from 5 to 55°C, utilizing a thermocycling machine (SD Mechatronic Thermocycler, Germany). Fracture resistance was assessed using a universal testing machine (Instron 3345 Series, UK) at 1 mm/min crosshead speed. Data were analyzed using One-way ANOVA, followed by Tukey’s post hoc test. The level of statistical significance was set at p<0.05.

**Results:**

The results indicated a significant difference in fracture resistance across groups (p<0.001). The control group exhibited the highest strength (935.12±114.52 N), followed by the Packable (926.79±229.36 N) and Injectable groups (923.29±110.28 N), with no significant differences. In contrast, the Bulk-Restorative and Bulk-Flowable groups had the lowest strengths (684.20±163.60 N and 616.08±132.54 N, respectively). Post hoc comparison showed significantly lower fracture resistance values in the bulk-restorative and bulk-flowable groups (p<0.001).

**Conclusion:**

Incremental placement of packable or injectable giomers restored fracture resistance to levels comparable to sound teeth, while bulk-fill techniques yielded inferior outcomes.

**Clinical significance:**

Clinicians frequently opt for bulk-fill restorative materials due to their efficiency and user-friendliness compared to traditional incremental layering techniques. However, evidence regarding the fracture resistance of giomers placed via different techniques remains limited. Injectable giomers represent a viable alternative, offering superior handling and precise adaptation while maintaining favorable mechanical properties. This study provides critical insights into optimizing placement strategies to balance clinical efficiency and biomechanical performance.

**Supplementary Information:**

The online version contains supplementary material available at 10.1186/s12903-025-06790-w.

## Introduction

With the growing focus on minimally invasive dentistry, direct restorations remain a viable restorative approach [1]. However, direct restorations pose challenges such as the time required for placement, ensuring proper adaptation, controlling polymerization shrinkage, and achieving adequate curing [2]. Moreover, the capacity of teeth to endure forces without fracturing or incurring damage indicates the strength of the entire restorative system. A clinically effective restorative system exhibits optimal stress transfer, enhanced functionality, and biological efficacy. Factors influencing treatment outcomes include initial defect size, preparation design, adhesive protocol, selected restorative material, and placement technique. Therefore, clinicians must carefully consider the interplay of all clinical variables to ensure the longevity of restorations [3–5].

The literature and clinical practice indicate that tooth integrity is significantly compromised in MOD cavities due to the loss of marginal ridge support, which increases the risk of fractures [6]. The selection of the direct restoration approach can impact clinical performance in the restoration of MOD cavities. Fracture resistance testing serves as a key indicator of the success of restorative systems. This testing has predominantly been applied to endodontically treated teeth, inlay-restored teeth, and situations where fiber reinforcement is considered beneficial, particularly in extensive cavities [7–9]. In the meantime, studies have been conducted to different degrees regarding the ability of resin composites to restore MOD cavities [10].

Resin composites have experienced considerable advancement over the years. Nevertheless, manufacturers continue to enhance these materials to allow clinicians to optimize their properties. To streamline and accelerate restorative procedures, resin composites with diverse consistencies and rheological characteristics are continually being launched in the market [11]. The incremental layering of resin composites is recognized as a standard procedure to reduce polymerization shrinkage stresses and increase marginal and structural integrity [12]. Injectable resin composite materials have gained prominence, offering improved adaptation and wettability of cavity floors and walls while retaining sufficient strength and mechanical properties essential for clinical success under the demanding conditions of the oral environment [13]. Similarly, bulk-fill materials were introduced to enhance the depth of cure and control shrinkage [14]. Furthermore, bulk-fill materials are available in both restorative/packable and flowable formulations, granting clinicians flexibility to select the most convenient approach for restorative procedures, optimizing ease, speed, force application, and stress distribution during polymerization, as well as subsequent functional performance [15, 16].

Although giomers are not novel to the market, their recent surge in popularity necessitates an investigation into their purported material versatility and potential for enhanced performance in clinically demanding scenarios [17]. Due to their favorable handling characteristics and sculptability, evaluating the mechanical suitability of giomers is critical, as clinicians may employ diverse placement techniques when restoring MOD cavities directly. Currently, there is a lack of evidence-based guidelines regarding the influence of placement technique and giomer formulation on the fracture resistance of maxillary premolars [18]. Therefore, clinically available, cost-effective giomers that claim versatility, efficient curing, and controlled polymerization shrinkage stresses and flow require thorough investigation.

This study evaluated the fracture resistance of maxillary premolars restored with injectable giomer versus bulk-fill giomer restorative material versus conventional, incrementally packed giomer after undergoing thermocycling. The null hypothesis of the study posited that the filling or placement technique does not affect the fracture resistance of giomer-restored MOD cavities in maxillary premolars.

## Materials and methods

Sample size analysis was conducted at MBU/RSU (Medical Biostatistics and Research Support Unit), Faculty of Dentistry, Cairo University, followed by gaining ethical approval for conducting this study from the Ethics Committee, Faculty of Dentistry, Ain Shams University (FDASU Rec EM 072411). Sample size calculation was based on a previous study [19] that assessed the fracture resistance mean values of three different resin-based restorative materials or giomer. Using a One-way ANOVA test, 5% statistical significance level, 80% power, and an effect size of 0.542, the total sample size calculated was 36 teeth. The total sample size was increased to 70 teeth to account for non-parametric compensation. With an allocation ratio of 1:1:1, each group comprised 14 teeth. The sample size calculation was performed using G*Power Software Version 3.1.9.7 [20].

This in-vitro study involved 70 sound maxillary premolars, with 56 teeth prepared for standardized MOD cavities and 14 retained as an intact control group. All teeth were extracted from patients aged 18–25 years for orthodontic purposes. After extraction, teeth were cleaned to remove residual soft tissues and stored in saline solution for one month. Only double-rooted maxillary premolars were included, while those with non-conforming root configurations were excluded. Teeth were examined under magnification (5x, Univet Loupes, Italy) to exclude specimens exhibiting cracks, craze lines, demineralization, or visible structural alterations.

**Fig. 1:**
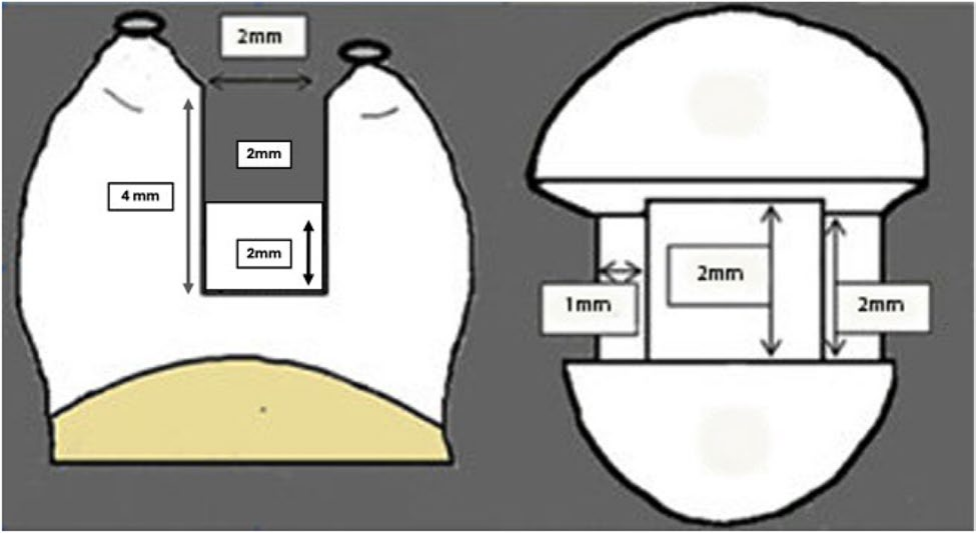
Tooth Preparation (MOD Cavity)

The standardized cavity design adopted in this study was a non-beveled MOD complex Class II cavity preparation measuring approximately 2 mm buccolingually, not exceeding one-third of the intercuspal distance. Cavities were prepared using an H7.FG.008 (330) Komet USA bur. The floor depth was maintained at 2 mm ± 0.2 mm at the central part of the cavity, while the full depth at the proximal portion (with a step) measured 4 mm ± 0.2 mm. Proximal box preparation was performed using a straight fissure bur (H21.314.012, Komet USA) operated by a high-speed handpiece (T3 Racer, Dentsply Sirona, NC, USA) under copious air/water spray (Fig. 1) [21]. During preparation, measurements and dimensions were verified using a graduated periodontal probe. Photographic documentation of all procedural steps was performed concurrently. Also, a single operator performed all cavity preparation phases for all teeth, while another performed the restoration phase from start to finish.

Following preparation, teeth were randomly allocated into four experimental groups and one control group using the Random.org software, which generated a true random sequence to assign each of the 70 teeth to one of the five groups (*n* = 14 per group). All materials used in this study were SPRG-containing, resin-based giomers with distinct rheological properties and curing protocols. All material details are shown in Table 1.

**Table 1:** Restorative materials used in this study, including compositions and manufacturers

Material	Composition	Manufacturer
Beautifil II LS (Low Shrinkage) *Shade A2*	Urethane diacrylate, Bis-MPEPP, Bis-GMA, TEGDMA, Polymerization initiator, Pigments, etc. S-PRG filler (fluoro- boroalumino- silicate glass) Filler %: 82.9 wt% (68.6 vol%) with a particle size of 0.01-4, mean 0.8	SHOFU INC., Kyoto, Japan **REF PNY2271**
Beautifil Flow Plus X Injectable (Injectable Bioactive Giomer) *Shade A2*	Bis-GMA, Bis-MPEPP, TEGDMA S-PRG fillers based on aluminofluoroboro-silicate glass, Al_2_O_3_ (64 wt%) 0.8 μm	SHOFU INC., Kyoto, Japan **REF YN2302**
Beautifil-Bulk Restorative (High viscosity bulk-fill giomer) *Shade Universal*	Bis-GMA, UDMA, Bis-MPEPP, TEGDMA Fluorosilicate glass (87.0 wt%,74.5 vol%)	SHOFU INC., Kyoto, Japan **REF PN2034**
Beautifil-Bulk Flowable (Low viscosity bulk-fill giomer) *Shade Universal*	Bis-GMA, UDMA, Bis-MPEPP, TEGDMA fluorosilicate glass (72.5 wt%,51.0 vol%)	SHOFU INC., Kyoto, Japan **REF PN2030**
BeautiBond Xtreme	Phosphate ester monomer, Carboxylic acid monomer, Dithiooctanoate monomers, Novel Acid Resistant Silane coupling agent, Methacrylate resin (Bis-GMA, TEGMA) Acetone & Water, Photoinitiator	SHOFU INC., Kyoto, Japan **REF PNY2449**

*Bis-GMA* Bisphenolglycidyl methacrylate, *TEGDMA *Triethylene glycol dimethacrylate, *UDMA* Urethane dimethacrylate, *Bis-MPEPP* 4-methacrylate polyethoxyphenyl propane

The experimental groups were designated as follows:

- Group I (Control): Sound maxillary premolar teeth (no intervention).
- Group II (Packable Giomer): Beautifil II LS (Shofu, Shade A2) placed in 2 mm increments.
- Group III (Injectable Giomer): Beautifil Flow Plus X (Shade A2) applied via syringe.
- Group IV (Bulk Restorative Giomer): Beautifil-Bulk Restorative (Shade Universal).
- Group V (Bulk Flowable Giomer): Beautifil-Bulk Flowable (Shade Universal) with a 2 mm occlusal capping layer.

Standardized procedures were followed for tooth isolation, matrix application, and bonding for all groups. First, the Tofflemire matrix band was positioned and tightened. The band was adjusted, ensuring a seal at gingival seats and margins. Then, selective etching of all margins was carried out using 37% phosphoric acid (META-Etchant, META-Biomed LTD, Korea) for 20 seconds. Afterward, rinsing was performed for double time of etching time, followed by necessary dryness. Next, BeautiBond Xtreme (Universal Adhesive, SHOFU INC., Kyoto, Japan) was actively applied in rubbing motion for 20 seconds. The adhesive layer was subsequently air-thinned in accordance with the manufacturer’s specifications. Subsequently, light curing was conducted for 20 seconds utilizing the 3M™ Elipar™ S10 LED Curing Light (3M ESPE, Deutschland, GmbH), with a verified output of 1200 mW/cm^2^.

### Giomer placement

The packable Beautifil II LS material was applied using the conventional incremental packing technique. The cavity was filled with successive 2 mm increments following the same matric and bonding protocol. Placement began at the gingival seat to ensure adaptation and gingival margin seal, followed by incremental advancement toward the proximal walls until the entire cavity volume was restored. The shaping of occlusal anatomy with a composite applicator/ball burnisher was comparable to that of other experimental groups.

For Group III, Beautifil Flow Plus X Injectable, the first step involved injecting a 2-mm increment, followed by an additional, filling the full depth of the cavity. The syringe tip was moved upward to fill the cavity depth, minimizing void formation. Occlusal anatomy was shaped using a composite applicator. Curing was subsequently conducted using the identical light-curing LED device. Further curing was conducted for the material bulk following the removal of the Tofflemire matrix band and setup. This post-matrix curing step was uniformly applied across all groups.

In accordance with the manufacturer’s guidelines for Group IV Beautifil-Bulk Restorative, giomer material was dispensed and adequately adapted, followed by curing in a single thick increment as indicated in 4 mm. Meanwhile, for the final Group V (Beautifil-Bulk Flowable), 4 mm cavity filling started at the gingival seat upwards, followed by an occlusal capping layer of 2 mm of Beautifil II LS. A periodontal probe was used to ensure sufficient thickness for the occlusal surface layer.

For all restorations, finishing was performed using a high-speed, yellow-coded stone/abrasive diamond (TR-13EF Yellow, MANI INC., Japan) operated by a high-speed handpiece (T3 Racer, Dentsply Sirona, NC, USA) with copious air/water spray. This was followed by a standard step of polishing using a OneGloss™ IC Inverted Cone (REF PN0183, Shofu Inc., Kyoto, Japan) operated for 15 s with maximum pressure limited to 0.3 N and speed within the 3000–10,000 rpm range under intermittent water spray.

### Thermocycling

All teeth were previously set in self-cure, acrylic resin blocks for testing (Acrostone, Dent Product, Egypt) with periodontal ligament simulation (elite HD + light body, Zhermack Italy) [22, 23]. In order to achieve this, root surfaces were immersed in melted wax (Crown wax BEGO GmbH & Co. KG) to a depth of 2.0 mm apical to the CEJ, resulting in a 0.2–0.3 mm thickness wax layer. Subsequently, acrylic resin blocks were fabricated using a plastic cylindrical mold of 25 mm. Following resin polymerization, the teeth were extracted from the cylinder, and the wax was eliminated from the root. The silicon material was injected into the cylinder, the tooth was reinserted, and the excess wax was removed using a scalpel blade. After completing all procedures, all teeth were exposed to 10,000 cycles of fluctuating 5–55 °C water baths using a thermocycling machine (SD Mechatronic Thermocycler, Germany). Specimens were steadily immersed at each temperature basin for 30 s each, with an interval of 10 s between both.

### Fracture resistance testing

Fracture resistance testing was performed using an INSTRON Universal Testing Machine (Instron 3345 Series, Instron Testing, MA, US). The test was conducted, and data were calculated and recorded utilizing Bluehill^®^ Universal computer software. A spherical load applicator with a diameter of 4 mm was centrally positioned on the occlusal surface of each premolar, situated between the buccal and palatal cusps, and aligned parallel to the long axis of the tooth. The test was conducted at a crosshead speed of 1 mm/min. The load was applied until failure, and the force at which the tooth was fractured was recorded in Newton as fracture resistance.

### Fracture mode analysis

Three observers evaluated fractured specimens (Canon EOS 700D Digital SLR with Sigma 105 macro lens) to identify the mode and pattern of failure. The pattern of failure detected was used to assign each specimen to one of two categories: favorable or unfavorable. Favorable failures exhibited repairable fractures above the cementoenamel junction (CEJ), which involved enamel, dentin, and/or restoration. On the other hand, catastrophic fractures below the CEJ that were beyond repair attempts were considered unfavorable failures. The numbers of each fracture were recorded, and subsequently, percentages were calculated for each experimental group.

**Fig. 2:**
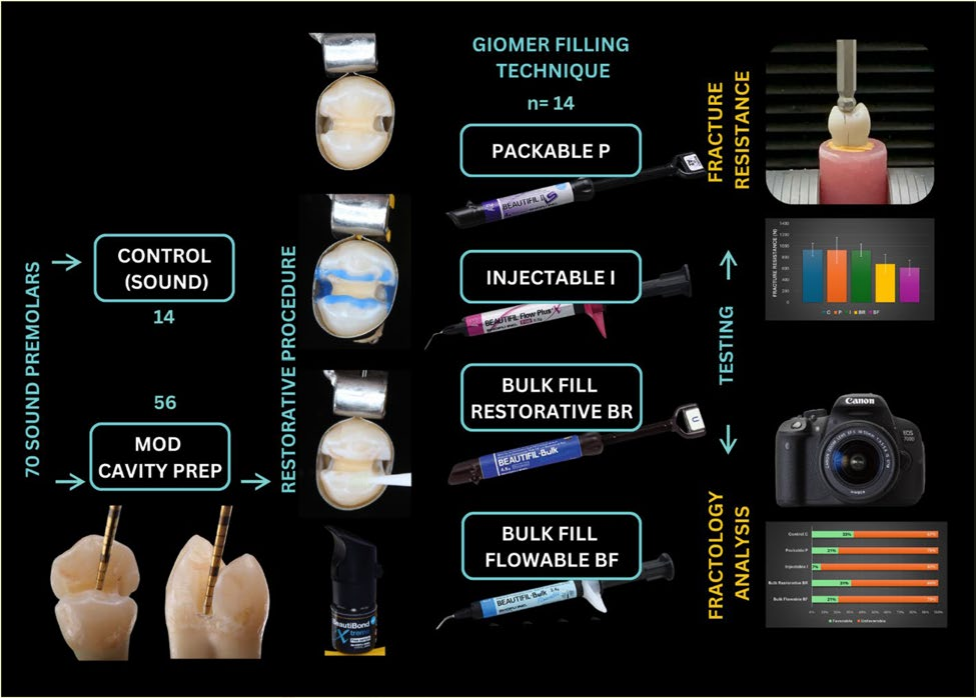
The study workflow outlines cavity preparation, restorative procedures, and classifications based on various giomer filling techniques, subsequently leading to fracture resistance testing and analysis of fracture modes

The entire experiment workflow is shown in Fig. 2*.*

### Statistical analysis

Numerical data were presented as mean with 95% confidence intervals and standard deviation (SD). Shapiro-Wilk’s and Levene’s tests were used to test for data normality and variance homogeneity, respectively. The data exhibited a normal distribution (*p* > 0.05) and homogeneity of variances across groups (*p* > 0.05). Analysis was conducted using one-way ANOVA, followed by Tukey’s post hoc test. The significance level was established at *p* < 0.05 for all tests conducted. Statistical analysis was conducted using R statistical software version 4.4.1 for Windows [24].

## Results

**Fig. 3:**
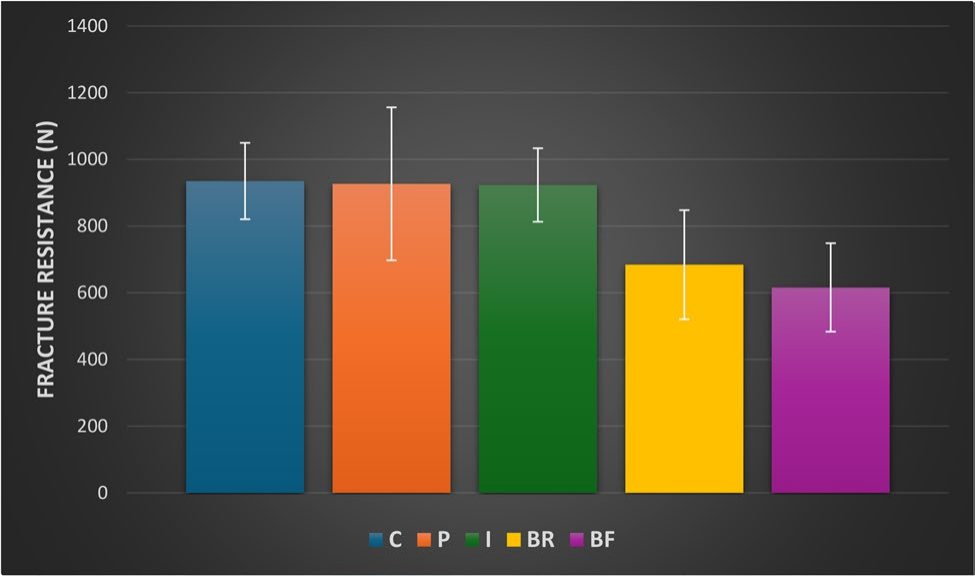
Bar chart showing mean and standard deviation values (error bars) of fracture resistance expressed in Newton (N)

The results highlighting mean, standard deviation, and intergroup comparisons are presented in Table 2. Results showed a significant difference between different groups (*p* < 0.001). The highest strength was recorded in the Control Group (935.12 ± 114.52 N), followed by the Packable group (926.79 ± 229.36 N), and then the Injectable group (923.29 ± 110.28 N), with no significant differences among these groups. The Bulk-Restorative group exhibited the lowest strength at 684.20 ± 163.60 N, while the Bulk-Flowable group demonstrated a strength of 616.08 ± 132.54 N. Post hoc pairwise comparisons indicated that the Control, Packable, and Injectable groups exhibited significantly higher strength values compared to the Bulk groups (*p* < 0.001). Mean and standard deviation values for fracture resistance results are presented in Figure 3.

**Table 2:** Fracture resistance values of the different experimental groups

Fracture resistance (*N*) (Mean ± SD)					f-value	p*
Control (C)	Packable (*P*)	Injectable (I)	Bulk Restorative (BR)	Bulk Flowable (BF)		
935.12 ± 114.52^A^	926.79 ± 229.36^A^	923.29 ± 110.28^A^	684.20 ± 163.60^B^	616.08 ± 132.54^B^	**14.16**	**< 0.001**

Values with different superscripts are significantly different

*significant (*p* < 0.05)

**Fig. 4:**
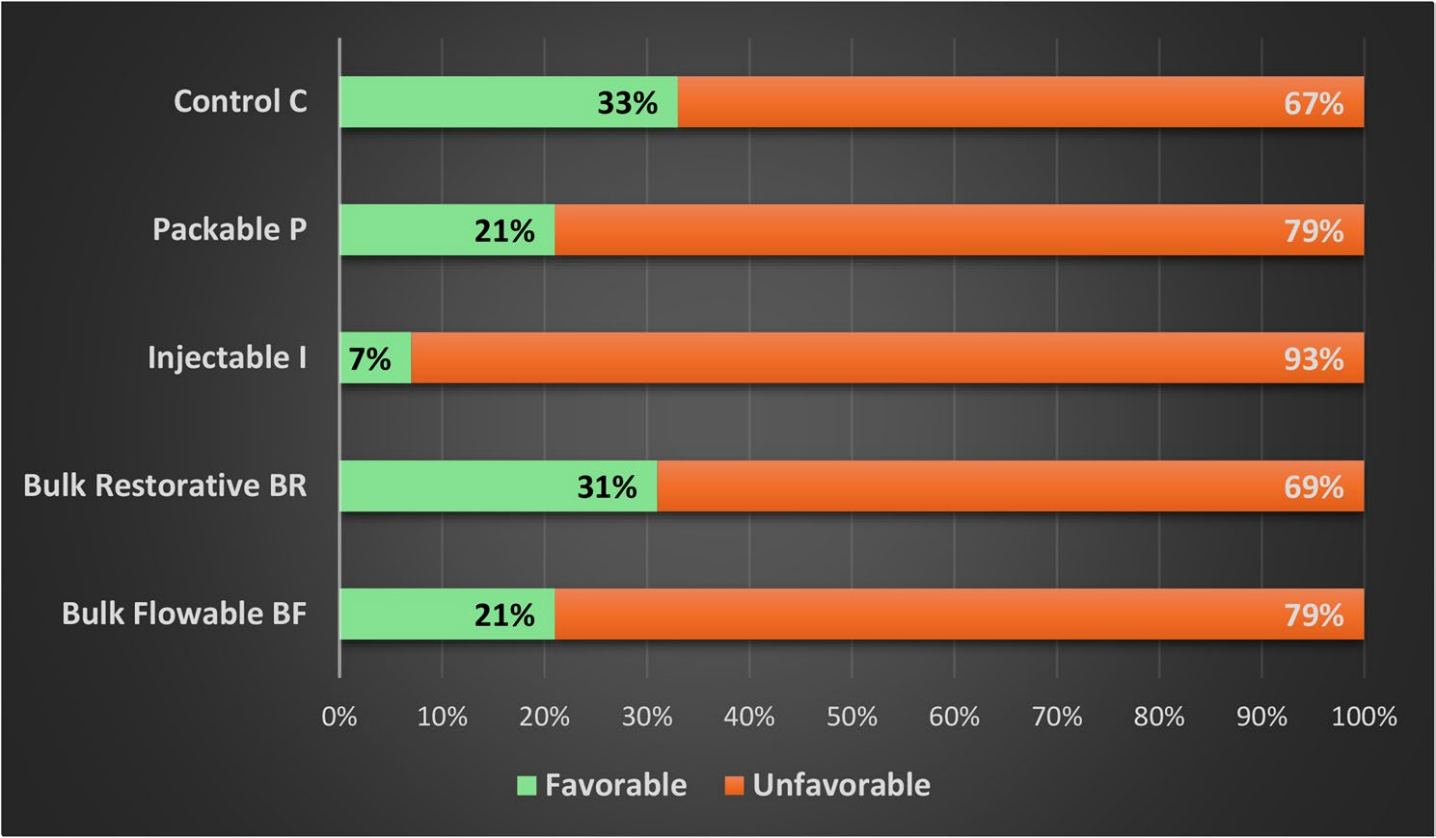
Stacked bar chart showing the results of fracture mode analysis for the experimental groups, expressed as percentages of favorable versus unfavorable fractures

Examination of specimens after fracture resistance testing revealed the following findings, as illustrated in Figure 4. The highest incidence of unfavorable failure was recorded in Group I Injectable (93%), with as minimal as 7% favorable fractures. Meanwhile, Group Packable (P) and Group Bulkfil Flowable (BF) recorded 79% unfavorable fractures, with only 21% favorable fractures. Finally, the Bulkfil Restorative and control groups recorded 67% and 69% unfavorable fractures, respectively.

**Fig. 5:**
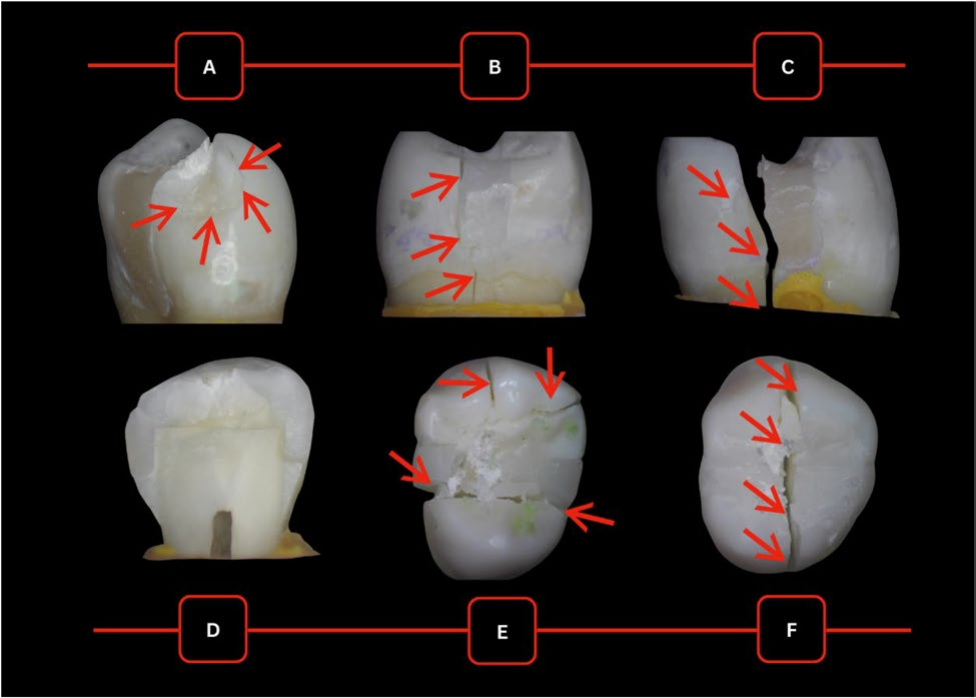
Exemplar images of fracture patterns across different groups:**a** evident chipping *in the specimen from Group II Packable*, **b** fracture along palatal cusp at the wall of restoration *in the specimen from Group III Injectable*, **c** palatal fracture running through restoration *in the specimen from Group II Packable*,**d** complete splitting of the tooth in two halves mesiodistally *in the specimen from Group V Bulk-Flowable*, **e** shattering of buccal and palatal cusps in multiple directions *in the specimen from Group IV Bulk-Restorative*, and **f** aggressive splitting of tooth in buccolingual direction *in Group V Bulk-Flowable*

Different images of fracture patterns from the experimental groups are demonstrated in Fig. 5. Most specimens showed a consistent pattern of vertical fracture, splitting the tooth into two halves at the junction of the restoration and tooth, with the fracture line almost always traveling along the palatal wall of the restoration and extending to the palatal third of the gingival seat before progressing into the root structure. Catastrophic irreparable fractures involve the restoration of the coronal tooth structure and root. The least common fracture pattern across all groups was minor to significant enamel chipping, with or without dentin involvement confined to the crown.

## Discussion

Manufacturing advancements have resulted in the development of materials designed to withstand the challenges and high stresses in the oral environment. Moosavi et al. [25] examined the impact of composite resin placement techniques on the success of restorations. Some studies indicate that bulk-fill techniques may produce less cuspal strain compared to incremental methods; however, this does not necessarily improve fracture resistance in MOD cavities with compromised cusps [26]. Conversely, Shefali et al. [27] posited that restoration quality depends on the material (resin composite or giomer) and the restoration technique, which affect fracture resistance. Therefore, this study investigated the conditions under which SPRG-containing, resin-based giomers optimize restoration success and examined various giomer formulations based on their rheological properties and curing requirements.

The mechanical performance of the tooth-restoration complex is assessed through fracture resistance testing on premolars, which are particularly susceptible to cusp fractures due to their anatomical shape, crown-root ratio, and crown volume. The findings of this study demonstrate a statistically significant difference in fracture resistance among the groups (f = 14.16, *p* < 0.001). Intact teeth (control) demonstrated the highest mean fracture resistance (935.12 ± 114.52 N), followed closely by the packable (926.79 ± 229.36 N) and injectable (923.29 ± 110.28 N) groups. These findings are consistent with those of Dalpino PH et al. [28], who reported that maxillary premolars restored with composite resin achieved fracture resistance statistically similar to that of sound teeth. This similarity may be attributed to the elastic deformation capabilities of these materials, which mimic the mechanical behavior of natural tooth structures. Notably, composite resin restorations predominantly failed via irreparable axial fractures, while failures in sound teeth were mainly repairable.

Post hoc pairwise analyses revealed no significant differences in fracture resistance among the injectable, control, and packable groups, suggesting that, despite overall group variations, these groups exhibit comparable performance. The injectable composite signifies an advanced category of high-strength flowable materials characterized by enhanced mechanical properties. These materials are appropriate for various direct anterior and posterior restorations, including areas subjected to high stress. Basheer RR et al. [29] corroborated these findings, demonstrating that injectable flowable composites exhibit flexural strength, hardness, roughness, and microleakage properties similar to those of nanohybrid packable composites. Their conclusions highlighted that the physical and mechanical properties of high-strength flowable composites are comparable to those of conventional composites, highlighting the necessity for dentists to comprehend material properties for informed selection.

Gerges et al. [30] found that packable and injectable materials exhibited comparable performance in small- and medium-sized mesio-occlusal cavities. In our study, the influence of cavity configuration was more pronounced due to the loss of both marginal ridges. This finding highlights the necessity of evaluating the clinical context and long-term durability in selecting restorative materials, as suitable choices may alter fracture patterns towards more favorable, repairable outcomes [30].

The bulk-fill group exhibited the lowest mean fracture resistance, likely due to the high filler content of giomer bulk restorative materials. High filler content can inhibit sufficient light penetration, leading to a reduced degree of conversion and incomplete polymerization. This is consistent with Tsujimoto et al. [31], who indicated that the depth of cure for bulk-fill materials is significantly impacted by light intensity. This observation contrasts with the findings of Ilie and Fleming [15], who reported that high-viscosity bulk-fill giomer materials exhibited superior micromechanical properties compared to conventional composite materials. Shimokawa et al. [32]. demonstrated that merely 10% of light penetrates a 4 mm-thick bulk material, indicating a direct correlation between radiant exposure and microhardness.

The distinctive composition of giomer, which integrates glass fillers with resin matrices, provides improved aesthetics and diminished polymerization shrinkage relative to conventional composites. Research indicates that pre-reacted glass in giomers can reduce polymerization shrinkage, thereby improving sealing at the tooth-restoration interface [33]. Hayashi et al. [34] assessed the sealing performance and morphology of bulk-fill materials (placed at 4–5 mm depths) using optical coherence tomography (OCT). Varying gap formations were observed in all tested light-cured bulk-fill composites, potentially elucidating the lower fracture resistance observed in our study.

This study is limited by its in vitro design, which may not fully capture the dynamic forces and variability present in a clinical environment. Although standardized MOD cavity preparations and thermocycling protocols provide control, they may not reflect the full spectrum of clinical conditions. Additionally, the reliance on extracted premolars and static loading tests limits the generalizability of the findings. Further research involving clinical trials and dynamic testing protocols is necessary to validate these findings.

## Conclusions

The study’s findings indicate that the incremental placement of packable giomer significantly improved fracture resistance, comparable to sound teeth. Although injectable giomer showed comparable strength, its higher occurrence of unfavorable fracture modes indicates potential long-term durability concerns. The bulk-fill variants did not adequately restore normal fracture resistance. The findings reject the null hypothesis, demonstrating that the filling technique has a significant impact on the mechanical performance of giomer-restored MOD cavities.

Clinically, while bulk-fill methods provide advantages in terms of ease of use and reduced placement time, the traditional incremental technique appears to be more effective for optimizing structural integrity in premolar restorations. The findings underscore the significance of meticulous material selection and technique consideration in restorative dentistry. Results must be interpreted cautiously, as in-vitro fracture resistance does not directly correlate with the clinical longevity of the restoration. Additional clinical research is necessary to confirm these findings and enhance comprehension of their implications in practical applications.

## Supplementary Information

Supplementary Material 1.

Supplementary Material 2.

## Data Availability

The data supporting the findings of this study will be made available upon request.

## Abbreviations

MOD: Mesio-occluso-distal
SPRG: Surface pre-reacted glass-ionomer
LS: Low shrinkage
Bis-MPEPP: 2,2-bis (4-methacryloxy poly-ethoxyphenyl) propane
Bis-GMA: Bisphenol a-glycidyl methacrylate
TEGDMA: Triethylene glycol dimethacrylate
UDMA: Urethane dimethacrylate
CEJ: Cementoenamel junction
OCT: Optical coherence tomography
