# Supplementary figures and images for: Influence of filling technique on fracture resistance of giomer-restored MOD-cavities
Source: BMC Oral Health. 2025 Sep 19;25:1415. doi: 10.1186/s12903-025-06790-w (PMC12449797; doi:10.1186/s12903-025-06790-w)

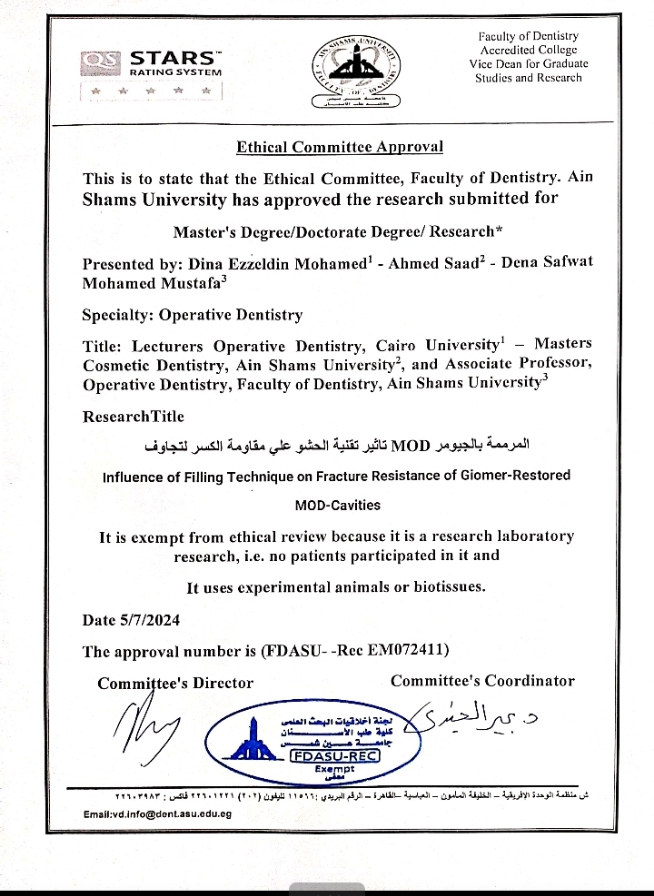

Supplement: Supplementary file 1 — Supplementary Material 1. [file 12903_2025_6790_MOESM1_ESM.jpg]
