# Supplementary material for: Influence of filling technique on fracture resistance of giomer-restored MOD-cavities
Source: BMC Oral Health. 2025 Sep 19;25:1415. doi: 10.1186/s12903-025-06790-w (PMC12449797; doi:10.1186/s12903-025-06790-w)
Supplement: Supplementary file 2 — Supplementary Material 2. [file 12903_2025_6790_MOESM2_ESM.pdf]

Ethical Committee Approval - Annex

This is to state that the Ethical Committee, Faculty of Dentistry. Ain Shams University has approved the research submitted for

Master's Degree/Doctorate Degree/ Research*

Presented by: Dina Ezzeldin Mohamed[1] - Ahmed Saad[2] - Dena Safwat Mohamed Mustafa[3]

Specialty: Operative Dentistry

Title: Lecturers Operative Dentistry, Cairo University[1] – Masters Cosmetic Dentistry, Ain Shams University[2], and Associate Professor, Operative Dentistry, Faculty of Dentistry, Ain Shams University[3]

ResearchTitle

تاثير تقنية الحشو علي مقاومة الكسر لتجاوف MOD المرممة بالجيومر

Influence of Filling Technique on Fracture Resistance of Giomer-Restored MOD-Cavities

It has been exempted from ethical review for the following reasons. It is standard practice at Oral & Maxillofacial Surgery Department at Faculty of Dentistry, Ain Shams University that all patients sign an informed consent which includes that his/her extracted teeth may be used in dental research purposes. Teeth then are used anonymously.

Date 27/3/2025

The approval number is (FDASU- -Rec EM072411) and date 5/7/2024

Committee's Director | **Committee's Coordinator**

لجنة أخلاقيات البحث العلمي
كلية طب الأسنان
جامعة عين شمس
FDASU-REC
Exempt
معفى
